# Efficacy and safety of EXOWALK® on electromechanical-assisted gait training: study protocol for randomized controlled trial

**DOI:** 10.1186/s13063-022-06660-8

**Published:** 2022-09-02

**Authors:** Chi-Yeon Lim, Mun Jung Ko, Jin Won Lee, Soo Kyung Bok, Nam-Jong Paik, Yeon Gyo Nam, Bum Sun Kwon

**Affiliations:** 1 Department of Biostatistics, School of Medicine, Dongguk University, Goyang, South Korea; 2 HMH Co. Ltd, Incheon, Republic of Korea; 3 Department of Rehabilitation Medicine, Chungnam National University College of Medicine, Chungnam, South Korea; 4 Department of Rehabilitation Medicine, Seoul National University Bundang Hospital, Seoul, South Korea; 5 Dongguk University Posture Science Institute, Dongguk University College of Medicine, Goyang, South Korea; 6 Department of Rehabilitation Medicine, Dongguk University College of Medicine, Goyang, South Korea

**Keywords:** Gait, Stroke, Exoskeleton device, Rehabilitation

## Abstract

**Background:**

High-intensity repetitive task-specific practice might be the most effective strategy to promote motor recovery after stroke, and electromechanical-assisted gait training represents one of the treatment options. However, there is still difficulty in clarifying the difference between conventional gait training and electromechanically assisted gait training.

**Methods:**

The study is a multicenter, randomized, parallel-group clinical trial for stroke patients. Three clinical research centers in Korea (Dongguk University Ilsan Hospital, Chungnam National University Hospital, and Seoul National University Bundang Hospital) will participate in the clinical trial and 144 stroke patients will be registered. Enrolled patients are assigned to two groups, an experimental group and a control group, according to a randomization table. In addition, patients are treated for half an hour (one session) five times a week for 4 weeks. Both groups carry out basic rehabilitation (central nervous system development therapy and strength exercise) and the experimental group executes robotic walking rehabilitation treatment, and the control group executes conventional gait rehabilitation treatment. The primary endpoint variable is the Functional Ambulation Category (FAC) that determines the degree of independent walking and is measured before, after, and after 4 weeks of treatment. Secondary endpoint variables are 11 variables that take into account motor function and range, measured at the same time as the primary endpoint variable.

**Discussion:**

There are still insufficient data on the effectiveness of electromechanical-assisted gait training for stroke patients and large-scale research is lacking. Thus, the research described here is a large-scale study of stroke patients that can supplement the limitations mentioned in other previous studies. In addition, the clinical studies described here include physical epidemiological analysis parameters that can determine walking ability. The results of this study can lead to prove the generalizable effectiveness and safety of electromechanical-assisted gait training with EXOWALK®.

**Trial registration:**

Clinical Research Information Service (CRIS), Republic of Korea KCT0003411, Registered on 30 October 2018

**Supplementary Information:**

The online version contains supplementary material available at 10.1186/s13063-022-06660-8.

## Administrative information

**Table:** 

Title	Efficacy & Safety of EXOWALK® on Electromechanical-assisted gait training: study protocol for randomized controlled trial.
Trial registration	KCT0003411 Clinical Research Information Service (CRIS) https://cris.nih.go.kr/cris/search/detailSearch.do/21809
Protocol version	Version 1.0
Funding	The work is carried out in the years 2018 to 2020, Korea Health Technology R&D Project through the Korea Health Industry Development Institute (KHIDI), funded by the Ministry of Health & Welfare, Republic of Korea (grant number: HI18C2324). The funder, KHIDI will have no role in the collection, manage-ment, analysis, and interpretation of data; writing of the report; nor the decision to submit the report for publication.
Author details	Department of Biostatistics, School of Medicine, Dongguk University, Goyang, South Korea HMH Co. Ltd, Incheon, Republic of Korea. Department of Rehabilitation Medicine, School of Medicine, Chungnam National University, South Korea Department of Rehabilitation Medicine Seoul National University Bundang Hospital, South Korea Dongguk University Posture Science Institute, Dongguk University College of Medicine, Seoul, South Korea Department of Rehabilitation Medicine, Dongguk University College of Medicine, Gyeongju, South Korea
Name and contact information for the trial sponsor	Not applicable; this trial does not have a sponsor

## Background

Gait training following a stroke has the potential benefits to increase walking speed as a result of training results in similar improvements in walking quality and economy [1]. For gait rehabilitation, highly repetitive practice restores gait function [2]. A number of rehabilitation robots consisting of treadmill and exoskeleton have been developed to assist people with brain lesions in walking training, mainly in Europe and the USA, and some have been commercialized. Automated electromechanical gait training devices were developed to reduce dependence on therapists [3].

Electromechanical-assisted gait training that requires repetitive tasks can improve the neuro-plasticity with motor learning focus on the reorganization of brain tissue, resulting in better balance and faster gait speed [4]. Seven papers concluded that the group that received the walking rehabilitation robot treatment in the 2017 Cochran review was more effective than the group that received the conventional gait rehabilitation treatment, and six papers did not [5]. Many of evidence suggests that high-intensity repetitive task-specific practice might be the most effective strategy to promote motor recovery after stroke, and therefore, electromechanical-assisted gait training represents one of the treatment options [6]. The previous study of this device revealed that electromechanically assisted gait training for 30 min each day for 5 days a week for a period of 4 weeks was as effective as gait training by a physical therapist [7]. A recent study reported the effectiveness on gait function of electromechanically assisted gait training with chronic stroke patients [8].

Conventional gait training conducted by a physical therapist was a standard method of gait training for stroke patients. After the introduction of robot rehabilitation, many studies investigated the effectiveness of electromechanically assisted gait training by comparing it with that of conventional gait training [5]. However, many studies still have difficulty to clarify the difference between conventional gait training and electromechanically assisted gait training and besides presented one of the reasons for the limitation of insufficient participants [9–11]. Now we need to conduct a large-scale randomized design to determine the effectiveness of electromechanical-assisted gait training by comparing it with physical therapist-assisted conventional gait training.

EXOWALK® (HMH Co., HR-01, A67020.02, Grade 2, South Korea) is a recently developed electromechanical exoskeleton-assisted gait training device. This design provides a stable and firm standing ability with little chance of falling and obviates the need for an additional cane or walker compared with currently popular exoskeletons. Such designs are user-friendly without the need for a harness for weight support. Clinical data on the validity and safety of this technology needs to explore this new market. Accordingly, in order to establish clinical efficacy and safety grounds for health insurance registration of new medical technology certification and rehabilitation robot therapy, clinical trials for proving the safety and effectiveness of walking rehabilitation of the new medical device EXOWALK® are conducted for stroke patients with walking disabilities.

We have estimated the sample size by setting the change of gait function more conservatively and conduct a multi-center, large-scale randomized design to determine the effectiveness of electromechanical-assisted gait training with the EXOWALK®.

## Method/design

### Study design

This study on the efficacy and safety of electromechanical-assisted gait trainer EXOWALK ® (HR-01) is a multicenter, randomized, superiority, and parallel-group study with a 1:1 allocation ratio. All enrolled participants are patients with stroke. Three clinical research centers in Korea participate in this trial: Dongguk University Ilsan Hospital, Chungnam National University Hospital, and Seoul National University Bundang Hospital. The 144 participants who met the inclusion criteria were assigned 72 each to the test and control groups. If the study protocol is changed, the changes and reasons are delivered by e-mail to the research participating organizations. The changed research protocol is submitted to the IRB for deliberation.

A strategy to achieve adequate participant registration is to promote the installation of posters and hand-outs in hospitals for patients and visitors to view. Each participant provides informed consent before enrollment. The research director explains the contents of the consent form to the patient for 30 min using terms that are easy for the general public to understand. We identify the schedule of the participants and schedule a visitable date to prevent participants from dropping out and complete the follow-up. Also, when the date of the visit approaches, we inform the participants in advance.

### Screening

The screening is conducted based on data from patients who agreed in the agreement, patients who met the inclusion and exclusion criteria, and hospitals. The target sample size is 144 participants. The eligibility assessment was conducted by a rehabilitation doctor according to inclusion and exclusion criteria. The patients were included within 1 week after eligibility assessment.

### Inclusion/exclusion

The patients are screened for a stroke, 10 or more Mini-Mental State Examination (MMSE), Modified Ashworth Scale (MAS) grade 2 or lower, and standing with an assist. The following patients are excluded: (1) patients with poor cognition that are difficult to carry out instructions; (2) ataxia; (3) patients with MAS grade 3 or above; (4) patients with severe leg arthritis; (5) difficulty walking due to joint swelling of the lower leg; and (6) situations where other walking training cannot be performed.

### Compliance

Compliance was assessed clinically on a per patient per visit.

$$\mathrm{Compliance}\left(\%\right)=\frac{\mathrm{Actual}\ \mathrm{number}\ \mathrm{of}\ \mathrm{treatments}}{\mathrm{Planned}\ \mathrm{number}\ \mathrm{of}\ \mathrm{treatments}}\times 100$$

Records of the study medical device used, the number of treated, and intervals between visits are kept during the study. The number of treated is confirmed by counting at each visit and by cross-verification with the researcher. The staff member responsible for study supply handling is responsible for device accountability. Medical device accountability is recorded on a form not accessible to other staff members participating in the assessment of patients.

In addition to the routine monitoring procedures, a Good Clinical Practice (GCP) Quality Assurance audit is initiated and the purpose of audits is to evaluate compliance with the principles of GCP, international and local regulatory requirements, and the study protocol.

### Data collection and management

This study uses a newly created paper case report form (CRF) before the start of clinical trials according to the study protocol. The clinical research coordinator (CRC) of the clinical research center is responsible for CRF writing. After creating a CRF for all participants, data coding is performed.

Two independent entry staff enter into different databases, respectively. Check whether the entered data match using the verified SAS program (V.9.3 or later version), and if it does not match, check the data against the CRF to modify the data. Before entering into the database, set a minimum input rule and train the entry staff. Input rules can help improve the reliability of the input data by minimizing input errors that may occur when typing, and the entry staff is entered in line with the data entry standard operating procedures (SOP). If electronic data transmission is required, the DataBase Programmer (DBP) delivers the annotated CRF and table schema to the researcher.

For the protection of personal data and data security of the participants, physical security is maintained to prevent leakage of collected personal data. All participants’ paper CRFs and electronic documents are stored in a secure, access-controlled space or a locked file cabinet. Documents are stored and managed for 5 years after the completion of clinical trials.

### Randomization and blinding

This study is a multicenter, parallel-group, and single-blind trial. For this clinical trial, participants who meet all of the participants’ inclusion/exclusion criteria and agree to participate in this study are assigned to two groups, an experimental group and a control group, according to a randomization table. Randomization is performed by an independent statistician using a random number generator computerized by the block randomization method in SAS version 9.4 (SAS Institute Inc. Cary, NC, USA) or later. A separate randomization file is created for each research institute. Outcome evaluators were blinded for reducing the bias. In order to increase reliability by minimizing measurement error, the physical therapists with more than 5 years of experience conducted interventions and evaluations. At enrollment, we instructed patients not to reveal their allocation arm to the outcome evaluator. The blinding was achieved by allocating the tasks of study device handling and dispensation to an independent study team member who was unblinded and who was provided with the randomization schedule.

Randomization plans may be viewed in case of emergency such as a serious adverse event (SAE) when the blind should be removed for any participant. In this case, we will describe the reasons why randomization should be released, the procedure, the documentation required, the series of treatments, and the evaluation of the participants.

### Treatment and assessment

**Fig. 1:**
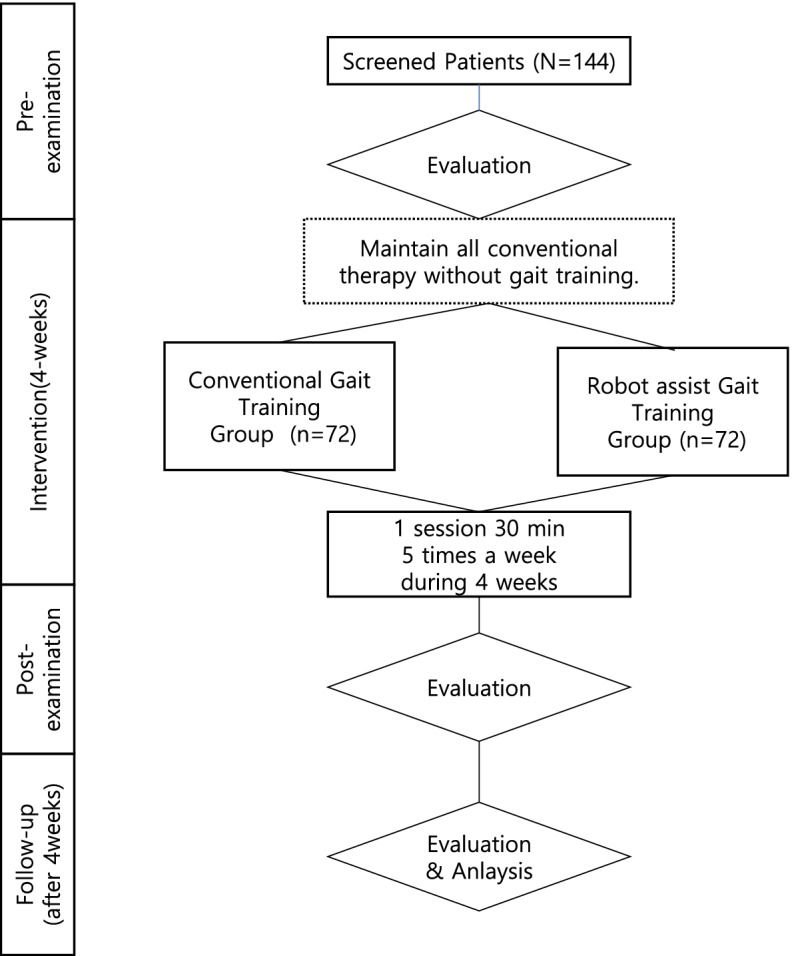
Flow chart for the protocol

Treatment and assessment were done by different physiotherapists with 5 years or more of experience, to increase the reliability by minimizing the measurement error. The experimental group received electromechanically assisted gait training with EXOWALK and the control group received conventional gait training by therapists (Fig. 1). Conventional gait training consisted of physical therapist-assisted gait training by verbal command and physical contact. Patients in both groups were given 30 min of training per session, five times per week for 4 weeks (Fig. 1). Both groups continued to have other physical and occupational therapy.

The intervention presented in this study, electrochemical-assisted gait training, is a treatment that has already been verified and applied clinically and is currently applied to stroke patients in research institutes. There are no expected side effects from academic research to verify the effectiveness of electromechanical-assisted gait training from various angles. However, due to the nature of the robot, a large amount of treatment is performed compared to conventional training, which can lead to weak muscle spasms and fatigue. To prevent this, training is conducted with sufficient rest according to the instructions of a professional therapist.

#### Existing rehabilitation treatment

All patients in both groups undergo the existing rehabilitation treatment. The existing rehabilitation consists of neurodevelopmental treatment (NDT) and strength training. NDT is a rehabilitation treatment that induces the balance and control of sitting and standing postures by activating the reflex action of the paralyzed lower limb using the Bobath technique. Strength training is a mobility exercise to increase the range of motion of the paralyzed muscles and strength training to improve strength.

#### Electromechanical-assisted gait training

As a treatment performed in the experimental group, the patients perform the electromechanical-assisted gait training in addition to the existing rehabilitation treatment. The medical device is EXOWALK® (HR-01), a rehabilitation robot for the lower limbs under clinical trials.

#### Conventional gait training

As a treatment performed in the control group, the physiotherapist guides and walks the patient while assisting on the side or back of the participants.

### Outcome measurements

In this study, the demographic and clinical characteristics of the participants are measured and documented after screening. Demographic information record gender, date of birth, height, weight, and joint problems or not. And clinical characteristics record a total of five screening criteria. Screening criteria are the name of the diagnosis, cause of the disabilities (brain infarction, cerebral hemorrhage), the paralysis side (Rt., Lt., both, quadriplegia), the possibility of expression of intention (standard: MMSE 10 or higher), and the lower limb spasticity MAS score (standard: MAS grade 2 or lower).

#### Primary outcome

The Functional Ambulation Category (FAC) is the primary endpoint for determining the existence of independent walking through a concise level assessment. Primary endpoints are evaluated once in the baseline (pre-treatment), 4 weeks after the baseline (post-treatment), and 4 weeks after the last treatment (follow-up), a total of three assessments (Table 1). FAC is a variable that was evaluated by dividing by 1 to 6 depending on the degree of need for assistance when walking, and FAC accounts for more than 55% as a result of the validity evaluation of the research paper on the rehabilitation robot in the 2017 Cochrane review. FAC level ranges from level 1 in “Nonfunctional” to level 6 in “Independent Level and Non-Level Surfaces.”

**Table 1:** Content of outcome measurement

Treatment number							
Outcome measure	Pre	1w	2w	3w	4w	Post	F/U
Primary outcome							
FAC	X					X	X
Secondary outcomes							
RMI	X					X	X
Walking velocity	X					X	X
Waking capacity	X					X	X
MI	X					X	X
Berg Balance Scale	X					X	X
MBI	X					X	X
Step counting	X					X	X
Borg scale	X					X	X
Swing time asymmetry	X					X	X
Step length asymmetry	X					X	X
Propulsion asymmetry	X					X	X

*Pre* pre-treatment, *1w* five times per week for the first week, *2w* five times per week for the 2nd week, *3w* five times per week for the 3rd week, *4w* five times per week for the 4th week, *Post* post-treatment, *F*/*U* follow-up, *FAC* Functional Ambulation Category, *RMI* Rivermead Mobility Index, *MI* Motricity Index, *MBI* Korean version of the Modified Barthel Index

#### Secondary outcomes

Secondary endpoints are Rivermead Mobility Index (RMI), 10-m walk test (walking velocity), 6-min walk test (walking capacity), Motricity Index (MI), Berg Balance Scale, the Korean version of the Modified Barthel Index (K-MBI), step counting, Borg scale, swing time asymmetry, step length asymmetry, and propulsion asymmetry. These evaluations are conducted once on the baseline (pre-treatment), 4 weeks after the baseline (post-treatment), and 4 weeks after the last treatment (follow-up), a total of three assessments (Table 1).

The second endpoints have a total of 11 assessments. First, RMI evaluates motor skills, consisting of 15 questions step by step, depending on the level of bed rotation to running. A total of 15 questions are scored 1 point if yes or 0 if no, and the total sum of the questions is used as a result of the evaluation. Second, walking velocity is a 10-m walk test that measures the speed during 10-m walking, and the unit is meter per second (m/s). Similarly, the third walking capacity is a 6-min walk test that measures the distance that can walk for 6 min, and the unit is meter (m). The fourth MI is an item to be evaluated in a 1 to 99 point system by measuring the lower leg force level from the ankle to the knee. These assessment items consist of three questions, each with a score of 0/9/14/19/25/33 and the total sum of the scores as a result of the evaluation. The fifth Berg Balance Scale is an item that evaluates balance ability by 0 to 56 points. There are 14 questions in total, and each question is scored from 0 to 4 points and the total sum of the scores is used as the result of the evaluation. The sixth K-MBI evaluates the independence of daily activities from 0 to 100 points. This assessment item consisted of 10 questions and, similarly, the total sum of the scores is used as a result of the evaluation. The seventh step counting measures the total number of steps performed by the patient during the specified walk-through time using the same mechanical counter by the three institutes. The measured number of steps is then used as a result of the assessment. The definition of a walk is the number of times a foot touches the ground again. The eighth Borg scale uses Borg’s rating of perceived exertion. Perceived exertion is defined as how much effort it takes to move or exercise. This item evaluates the cognitive level of the subjective effort intensity of the participants in the exercise from 0 (no breathlessness at all) point to 10 (maximal) points. Lastly, the three evaluation items are measured by motion analysis devices. There are swing time asymmetry analyzing gait phase, step length asymmetry analyzing gait phase, and propulsion evaluation item analyzing propulsion.

In addition, 4 weeks after baseline (post-treatment), patients in the experimental and control groups are questioned on the usability assessment of the electromechanical-assisted gait training. Also, the therapist who gave the treatment is questioned. The goal of this assessment is to verify and supplement the assessment items by verifying the usability level of various types of walk-through electromechanical-assisted gait trainers and verifying the reliability and validity of the usability assessment.

Finally, during the follow-up evaluation, the patients receive a follow-up assessment questionnaire to determine if the patients are treated for a period of 4 weeks of follow-up observation.

### Motion analysis devices

In the evaluation of research, there are two types of motion analysis devices to be used.

HumanTrack (Rbiotech, 1806A_DA004_H1FS), a gait analysis system, is a machine capable of performing walking analysis only at a distance of 5–7 m without space restriction, and even a seriously ill patient using an assist instrument can be measured.

Freemed (Rbiotech, 1807A_MX20321) is a foot pressure measurement system that allows patients and ordinary people to evaluate static and dynamic foot pressure, walking, and balancing abilities. In the static inspection, load ratios in the medical/lateral and anterior/posterior directions can be analyzed by the distribution of the pressure in the sole, and in the dynamic foot low-pressure inspection, not only temporo-spatial parameters such as the ratio of the foot angle standing device (instance phase) and the swing phase, but also walking pressure, average visibility, and walking force are provided.

### Safety assessment

The physical content of the clinical alteration is reported by the auditors, practitioners, and patients at each visit. All indication, data of onset, and period are recorded.

### Sample size

The primary endpoint is the FAC difference before and after training. Data were available on this parameter from a previous trial (Hiroko Watanabe et al. 2014). Hiroko Watanabe et al. show a study on the improvement of stroke walking ability using the hybrid assisted limb (HAL), a representative device of the walking rehabilitation robot. According to the reference, the mean change in existing medical devices was 0.54 and 1 in the test group and HAL group. The newly developed medical device for use in this study was expected to produce about 25% better results in performance than in the HAL group, assuming a variation of 1.25. Therefore, it was assumed that the difference between the variation in the FAC of the test medical device and the comparator device was 0.71 and that the standard deviation was conservatively approached to assume the largest value of 1.4. Two-sided tests were used for sample size estimation. The number of participants per group to achieve overall power of 80% under the significant level of 0.05 is 65. To allow for a possible 10% dropout rate, 144 people were registered per group with 72 people.

### Statistical analysis

Categorical variables such as gender, joint problem disorder, the cause of the disability, and paralysis side in demographic and clinical characteristic information will be presented as *n* (%). The pre-homogeneity will be analyzed by the chi-squared test or Fisher’s exact test. Continuous variables such as age, height, weight, the possibility of expression of intention, and the lower limb spasticity MAS score will be expressed as mean ± standard error (SE) and range. The pre-homogeneity will be analyzed using Student’s *t*-test or Wilcoxon’s rank sum test depending on whether the normality assumption is satisfied.

For all continuous variables of primary and secondary outcomes, the post-treatment measurement results will present the technological statistics, the observed number of patients, mean, SE, and range and compare the differences between groups using Student’s *t*-test or Wilcoxon rank sum test depending on the satisfaction of the normality assumption. The comparison analysis of differences before and after treatment in the group will be conducted using either the paired *t*-test or Wilcoxon’s signed tank test depending on the satisfaction of the normality assumption. In analyzing continuous variables, analysis of covariance (ANCOVA) will be performed after setting the variables as covariates when there are variables that seem to require control in the pre-homogeneity results for demographic and clinical characteristics.

Additional details regarding subgroup and adjusted analyses and definition of analysis populations are available in the clinicaltrials.gov (KCT0003411) in the study summary.

All data will be analyzed using SAS version 9.4 or later. All statistical tests will be two-sided, and the level of significance was set at 0.05.

### Compensation plans

In the event of damage or damage directly related to this clinical trial, the clinical trial manager will be responsible for providing compensation for the damage following the victim compensation regulations. In the case of side effects, the victim will be treated with a known treatment method. If an adverse event occurs due to a clinical trial, it will be treated properly until the subject recovers and compensated for the damage caused by clinical trial medical devices in accordance with the regulation for clinical trial victim compensation.

## Discussion

Automated electromechanical gait training devices have been developed and their effectiveness has been proven [12, 13]. However, other reports documented similar or superior effects of robot-assisted therapy in combination with conventional physiotherapy versus conventional therapy alone on gait recovery, especially in patients with sub-acute stroke [14, 15]. This clinical study aims to certify a recently developed medical device and demonstrates the safety and efficacy of EXOWALK, an electromechanical-assisted gait trainer, in stroke patients.

There are still sufficient papers on the effectiveness of electromechanical-assisted gait training for domestic patients and large-scale research is lacking. Thus, the research described here is a large-scale study of domestic patients that can supplement the limitations mentioned in other previous studies. In addition, the clinical studies described here include physical epidemiological analysis parameters that can determine walking ability. Therefore, it can prove that robotic therapy is effective for walking. The clinical is a multicenter study for domestic patients conducted by all three institutions in a thorough random sampling system, so it will be possible to prove its generalizable effectiveness and safety.

## Trial status

The clinical trial protocol is registered in the Clinical Research Information Service (CRIS) of the Republic of Korea. CRIS has joined the WHO International Clinical Trials Registry Platform (ICTRP) as the world’s 11th representative primary registry in Korea. Research information registered in CRIS is disclosed on the web in real time from the time of approval of the manager and is regularly transmitted to WHO ICTRP at regular intervals to foreign researchers and the public. The recruitment of patients was completed in June. The last patient registration date is 29 June 2020. A total of 144 people have been registered. Trial completion will be expected to be completed by the end of 2020. This protocol version is 1.4 (4 April 2019).

## Supplementary Information

**Additional file 1.** Study registraion information and protocol in CRIS.

## Data Availability

The datasets analyzed during the current study are available from the corresponding author on reasonable request.

## Abbreviations

CRIS: Clinical Research Information Service
KHIDI: Korea Health Industry Development Institute
MMSE: Mini-Mental State Examination
MAS: Modified Ashworth Scale
CRF: Case report form
CRC: Clinical research coordinator
SOP: Standard operating procedures
DBP: DataBase Programmer
SAE: Serious adverse event
NDT: Neurodevelopmental treatment
FAC: Functional Ambulation Category
RMI: Rivermead Mobility Index
MI: Motricity Index
K-MBI: Korean version of the Modified Barthel Index
HAL: Hybrid assisted limb
SE: Standard error
ANCOVA: Analysis of covariance
IRB: Institutional Review Board
