# Supplementary material for: Efficacy and safety of EXOWALK® on electromechanical-assisted gait training: study protocol for randomized controlled trial
Source: Trials. 2022 Sep 2;23:729. doi: 10.1186/s13063-022-06660-8 (PMC9438256; doi:10.1186/s13063-022-06660-8)
Supplement: Supplementary file 1 — Additional file 1. Study registraion information and protocol in CRIS. [file 13063_2022_6660_MOESM1_ESM.pdf]

Status : Approved
First Submitted Date : 2018/10/30 **Registered Date :** 2019/01/03
Last Updated Date : 2022/06/27

1. Background

CRIS Registration Number	KCT0003411
Unique Protocol ID	2018-08-026-001
Public/Brief Title	Multicenter Study on the Efficacy and Safety of Lower Limb Rehabilitation Robot.
Scientific Title	Multicenter Study on the Efficacy and Safety of Lower Limb Rehabilitation Robot using a newly-developed device Exowalk.
Acronym	The Efficacy of Lower Limb Rehabilitation Robot
MFDS Regulated Study	No
IND/IDE Protocol	No
Registered at Other Registry	No
Healthcare Benefit Approval Status	Not applicable

2. Institutional Review Board / Ethics Committee

Board Approval Status	Submitted approval
Board Approval Number	DUIH 2018-08-026-001
Approval Date	2018-10-30
Approval File	동국대병원_승인통지서_다기관.pdf
Institutional Review Board Name	Dongguk University Ilsan Hospital IRB
Institutional Review Board Address	27, Dongguk-ro, Ilsandong-gu, Goyang-si, Gyeonggi-do

Top
Bottom

Institutional Review Board Telephone	031-961-8405
Data Monitoring Committee	Yes<br>Donggukk Independent Data Monitoring Committee(DGIDMC)

3. Contact Details

- Contact Person for Principal Investigator / Scientific Queries

Name	BUMSUN KWON
Title	professor
Telephone	+82-31-961-7480
Affiliation	Dongguk University Ilsan Hospital
Address	814 Siksa-dong Ilsandong-gu Goyang-si Gyeonggi-do

- Contact Person for Public Queries

Name	Yeongyo Nam
Title	Researcher
Telephone	+82-31-961-8435
Affiliation	Dongguk University Ilsan Hospital
Address	814 Siksa-dong Ilsandong-gu Goyang-si Gyeonggi-do

- Contact Person for Updating Information

Name	Yeongyo Nam
Title	Researcher
Telephone	+82-31-961-8435
Affiliation	Dongguk University Ilsan Hospital
Address	814 Siksa-dong Ilsandong-gu Goyang-si Gyeonggi-do

4. Status

Top

Study Site	Multi-center Number of center : 3

Bottom

Overall Recruitment Status	Completed
Date of First Enrollment	2018-11-01 Actual
Target Number of Participant	144
Primary Completion Date	2020-08-13 , Actual
Study Completion Date	2020-08-13 , Actual

- Recruitment Status by Participating Study Site 1

Name of Study	Chungnam National University Hospital
Recruitment Status	Completed
Date of First Enrollment	2018-11-01 ,

- Recruitment Status by Participating Study Site 2

Name of Study	Seoul National University Bundang Hospital
Recruitment Status	Completed
Date of First Enrollment	2018-12-01 ,

- Recruitment Status by Participating Study Site 3

Name of Study	Dongguk University Ilsan Hospital
Recruitment Status	Completed
Date of First Enrollment	2018-12-07 ,

5. Source of Monetary / Material Support

- 1. Source of Monetary/Material Support

Organization Name	Ministry of Health & Welfare
Organization Type	Government

Top
Bottom

Project ID	HI18C2324

6. Sponsor Organization

- 1. Sponsor Organization

Organization Name	Dongguk University Ilsan Hospital
Organization Type	Medical Institute

7. Study Summary

Lay Summary	To evaluate the effectiveness of electromechanical-assisted gait training compared to conventional gait training on walking ability and symmetry in stroke patients.<br><Data management and statistical analysis><br>This research protocol has a potential risk that the possibility of exposing subjects' personal information in the process of collecting and registering medical records. To prevent this, both name and resident registration number are processed as encrypted information, so no personal information is disclosed.<br>For demographic and clinical characteristics, categorical variables are presented as frequency and percentage. They were analyzed for pre-homogeneity with Chi-squired tests. Continuous variables are presented as mean, standard deviation (SD), and range of minimum and maximum (Min, Max). In case of height and weight satisfying normality, pre-homogeneity was analyzed using student's t-test. For age not satisfying normality, Wilcoxon rank sum test was used.<br>All values of primary and secondary outcomes are presented as mean and SD (Mean ± SD). Within each group, values of pre and post-intervention changes were analyzed using paired t-test if normality was satisfied or Wilcoxon's signed rank test if normality was not satisfied. In addition, for comparison between pre and post-intervention values of test and control groups, student's t-test and Wilcoxon's rank sum test were performed.<br>Stroke duration was the most important factor that affected results. Subgroup analysis compared variations between subjects with stroke durations of 90 days or less (below 90 days) and those with 91 days or more (over 91 days) in the experimental group.<br>The effectiveness analysis of this clinical trial is performed on FAS (Full Analysis Set). All statistical analyses were performed using SAS version 9.4 or later. All statistical tests were two-sided and the level of significance was set at 0.05.<br>An interim analysis was not planned. Therefore, DMC did not consisted.<br><Quality assurance><br>Principal investigators will hold a researcher meeting or clinical trial initiation meeting for this clinical trial before the start of the clinical trial. In this meeting, detailed discussions on the clinical trial protocol, the implementation of the clinical trial procedure, and the preparation of the CRF, data entry, coding, data archiving will be held. Investigators who cannot attend these meetings, or who will later participate in clinical trials, should receive appropriate training by the sponsor or person authorized by the principal investigator.<br>-Monitoring<br>Designate a CRA in charge of this clinical trial to visit and monitor the laborator

Top

Bottom

	y before the start of the clinical trial and during the trial period. CRA explains the monitoring plan to the investigator before starting the clinical trial, and discusses the visit schedule with the research team of the conducting institution before each monitoring visit. In addition, during the monitoring visit, the CRA confirms that the investigator is conducting the clinical trial in accordance with the clinical trial protocol and related regulations. Matters found during monitoring should be properly discussed with the investigator and resolved. CRA will also be contacted and discussed with the investigator by means of communication such as telephone, fax, and e-mail. -Safety Monitoring If adverse events occur, report them to the IRB within 24 hours. In addition, the CRA organizes the SAE list and delivers it to the Principal Investigator(PI) and data manager(DM), and the DM checks whether all the SAEs provided are entered in the DB if it is not input, issue Data Clarification Forms (DCFs) to check if it is an SAE. After that, the Data Manager additionally enters the database. Even if it is not in the provided SAE list, if the SAE status is "Yes" among the entered data or if the contents judged to be SAE are written, the contents shall be forwarded to the CRA for confirmation.

8. Study Design

Study Type	Interventional Study
Study Purpose	Treatment
Phase	Not applicable
Intervention Model	Parallel
Blinding/Masking	Single
Blinded Subject	Investigator, Outcome Accessor
Allocation	RCT
Intervention Type	Medical Device
Intervention Description	This study on the efficacy and safety of Electromechanical -assisted gait trainer EXOWALK ®(HR-01) is a multicenter, randomized and parallel-group study. All enrolled subjects are patients with stroke. Three clinical research centers in Korea participate in this trial: Dongguk University Ilsan Hospital, Chungnam National University Hospital and Seoul National University Bundang Hospital. Participating institutions in the study received clinical research approval as a research proposal delivered by the responsible institution to the Institutional Review Board (IRB) of each institution.When the research plan was changed, the changes and reasons were delivered to the research participating institution by e-mail, and a request for reconsideration was made to the IRB with the changed research proposal. Strategies for achieving adequate participant enrolment is to promote the hospital, such as posters that patients and visitors can see. Each subject provides informed consent before enrollment. The research director explains the contents of the consent form to the patient for 30 minutes using terms that are eas

Top
Bottom

		y for the general public to understand.<br><br>The screening is conducted based on data from patients who agreed in agreement, patients who met the inclusion and exclusion criteria, and hospitals. The target sample size is 144 subjects.<br>This study is a multicenter, parallel-group and single blind trial. For this clinical trial, subjects who meet all of the subject inclusion/exclusion criteria and agree to participate in this study are assigned to two groups, an experimental group and a control group, according to a randomization table. Randomization is performed by an independent statistician using a random number generator computerized by the block randomization method in SAS version 9.4 (SAS institute Inc. Cary, NC, USA) or later. A separate randomization file is created for each research institute. Randomization plans may be viewed in case of emergency when the blind should be removed for any subject. In this case, we will describe the reasons why randomization should be released, the procedure, the documentation required, the series of treatments and the evaluation of the subject.<br>All patients in both groups are given 30 minutes (1 session) five times per week for four weeks. In addition, both groups perform basic rehabilitation (central nervous system development therapy and strength exercise) and the experimental group performed Electromechanical -assisted gait training with EXOWLK, and the control group performed conventional gait rehabilitation treatment. However, the presence or absence of existing rehabilitation treatments is different for outpatients. Therefore, if an outpatient was performing no existing rehabilitation treatment, proceed with the clinical test without existing rehabilitation and keep it in place if the existing rehabilitation treatment was being performed.<br><br>All patients in both groups undergo the existing rehabilitation treatment. The existing rehabilitation consists of Neurodevelopmental Treatment(NDT) and strength training. NDT is a rehabilitation treatment that induces the balance and control of sitting and standing postures by activating the reflex action of the paralyzed lower limb using the Bobath technique. Strength training is a mobility exercise to increase the range of motion of the paralyzed muscles and strength training to improve strength.<br>Electromechanical -assisted gait training<br>As a treatment performed in the experimental group, the patients perform the Electromechanical -assisted gait training in addition to the existing rehabilitation treatment. The medical device is ‘EXOWALK®(HR-01), a rehabilitation robot for the lower limbs under clinical trials.<br>As a treatment performed in the control group, the physiotherapist guides and walks the patient while assisting on the side or back of the subject.
Number of Arms		2
Arm 1	Arm Label	The experimental group
	Target Number of Participant	72
	Arm Type	Experimental

Top

Bottom

	Arm Type	
	Arm Description	Received electromechanical-assisted gait training with Exowalk®<br>30 minutes a day, 5 days a week for 4 weeks<br>As a treatment performed in the experimental group, the patients perform the Electromechanical -assisted gait training in addition to the existing rehabilitation treatment. The medical device is 'EXOWALK®(HR-01), a rehabilitation robot for the lower limbs under clinical trials.<br>Because patient's tolerance and safety of electromechanical gait training compared to physiotherapy need to be considered, Exowalk® facilitates less than 1000 steps in 30 min with a velocity of 1.8 km/h according to initial evaluation, although its maximum velocity is 2.3 km/h. Patients in this study were recommended to receive the electromechanical exoskeleton-assisted gait training at a comfortable speed.
Arm 2	Arm Label	The control group
	Target Number of Participant	72
	Arm Type	Active comparator
	Arm Description	Received conventional gait rehabilitation treatment by therapists.<br>30 minutes a day, 5 days a week for 4 weeks<br>For subjects in the control group, the physiotherapist guided and walked the patient while assisting the subject on the side or the back.

9. Subject Eligibility

Condition(s)/Problem(s)		* (I00-I99)Diseases of the circulatory system<br>(I64)Stroke, not specified as haemorrhage or infarction<br><br>Patients with Stroke.
Rare Disease		No
Inclusion Criteria	Gender	Both
	Age	No Limit~No Limit
	Description	1) those who had a stroke<br>2) those who had a score of 10 or more in the Mini-Mental State Examination (MMSE)<br>3) those who had a Modified Ashworth Scale (MAS) Grade 2 or lower<br>4) those who could stand alone.
Exclusion Criteria		1) those with poor cognition that made it difficult to carry out instructions<br>2) those with ataxia that made unstable standing balance<br>3) those with spasticity MAS Grade 3 or above

Top
Bottom

	4) those with severe leg arthritis<br>5) those with difficulty walking due to joint problems of the lower leg
Healthy Volunteers	No

10. Outcome Measure(s)

Type of Primary Outcome	Efficacy

- Primary Outcome(s) 1

Outcome	Functional ambulatory category (FAC), FAC was determined the existence of independent walking through a concise level assessment. Primary endpoints were evaluated once at baseline (pre-intervention) four weeks after the baseline (post-intervention), and four weeks after the last treatment (follow-up). FAC was evaluated by dividing the degree of needing for assistance when walking to 1 to 6. FAC level ranged from Level 1 for 'nonfunctional' to Level 6 for 'independent without help for non-level surfaces'.
Timepoint	before and after treatment, 4weeks follow up

- Secondary Outcome(s) 1

Outcome	Second endpoints had a total of 7 assessments. First, RMI was used to evaluate motor skills. It consisted of 15 questions step by step, depending on the level ranging from bed rotation to running. A total of 15 questions were scored. Each was scored 1 point if yes or 0 if no. The total sum was used as a result of the evaluation. Second, walking velocity as a 10mWT was used to measure the speed during a 10-meter walking. The unit was m/sec (meter per second). Similarly, walking capacity was evaluated with a 6MWT to measure the distance that one could walk for 6 minutes. The unit was m (meter). The fourth item was MI. It was evaluated as 1 to 99 points by measuring the lower leg force level from the ankle to the knee. Assessment items consisted of three questions, each with a score of 0/9/14/19/25/33. The total sum of scores was used as the result of the evaluation. The fifth item was BBS to evaluate the balance ability with 0 to 56 points. There were 14 questions in total. Each question was scored from 0 to 4 points. The total sum of scores was used as the result of the evaluation. The last two evaluation items were measured with motion analysis devices for those who could walk stable without aids. Swing time asymmetry and step length asymmetry were analyzed by HumanTrack (Rbiotech, 1806A_DA004_H1FS, South Korea) a gait analysis system which is capable of performing walking analysis at a distance of 5-7 m without space restriction. The swing time was calculated based on phase of gait begins when the foot first leaves the ground and ends when the same foot touches the ground again. The step length was calculated as the distance between the heel of the foot and the heel of the other foot. Each asymmetry value was calculated as the absolute value difference between the paretic side and the non-paretic side. The physical content of the clinical alteration was reported by auditors, practitioners, and patients at each visit. All indication, data of onset, and period were r
Timepoint	before and after treatment, 4weeks follow up

Top
Bottom

11. Study Results and Publication

Result Registered	Yes<br>Results Upload
Final Enrollment Number	144
Number of Publication	0
Results Upload	R.pdf
Date of Posting Results	22-06-27
Protocol URL or File Upload	Study_Protocol.png
Brief Summary	FAC showed significant improvement after 4 weeks intervention in both groups. Walking abilities showed significant improvement after intervention, but, walking symmetries did not in both groups. According to sub-group analysis of stroke duration of 90 days, FAC and walking abilities in both groups showed significant improvement in subacute group compared to chronic group. However, walking symmetries did not show any significant changes in subacute and chronic group. Swing time asymmetry in the experimental group showed significant improvement in chronic group while it did not in the control group.

12. Sharing of Study Data(Deidentified Individual-Patient Data, IPD)

Sharing Statement	Yes
Time of Sharing	2022. 1
Way of Sharing	Available on Request<br>(bskwon@dumc.or.kr)

목록으로 이동

Top

Bottom
